# Supplementary material for: Pinpointing a Mechanistic Switch Between Ketoreduction and “Ene” Reduction in Short‐Chain Dehydrogenases/Reductases
Source: Angew Chem Weinheim Bergstr Ger. 2016 Jul 13;128(33):9748–52. doi: 10.1002/ange.201603785 (PMC4988499; doi:10.1002/ange.201603785)
Supplement: Supplementary file 1 — Supplementary [file ANGE-128-9748-s001.pdf]

Angewandte Chemie
Eine Zeitschrift der Gesellschaft Deutscher Chemiker

Supporting Information

Pinpointing a Mechanistic Switch Between Ketoreduction and "Ene" Reduction in Short-Chain Dehydrogenases/Reductases

*Antonios Lygidakis[+], Vijaykumar Karuppiah[+], Robin Hoeven, Aisling Ní Cheallaigh, David Leys, John M. Gardiner, Helen S. Toogood, and Nigel S. Scrutton**

ange_201603785_sm_miscellaneous_information.pdf

Table of Contents

1. Supplementary Materials and Methods

2. Supplementary enzyme crystal structure discussion

3. Supplementary Figures

4. NMR traces

5. Supplementary Tables

6. Additional Crystal Structure Discussion

1. Supplementary Materials and Methods

General Reagents and Analytical Techniques

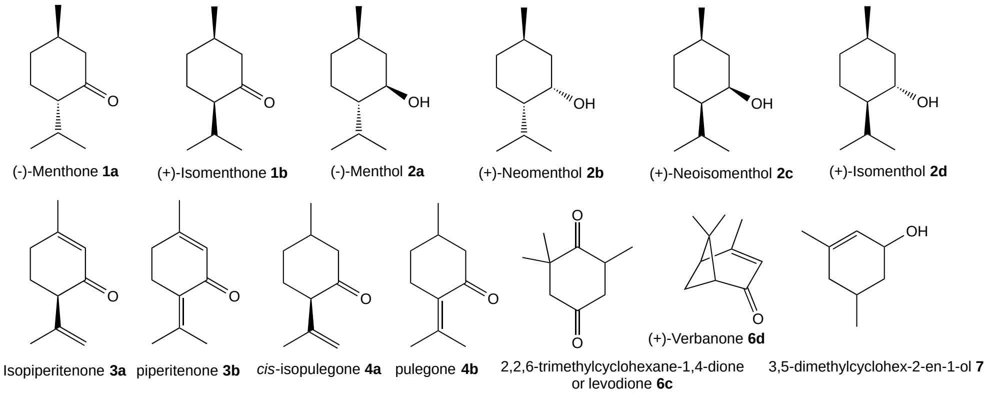

All chemicals and solvents were purchased from commercial suppliers, except where specified, and were of analytical grade or better. The synthesis of monoterpenoids **1a-b**, **2a-d**, **3a-b** and **4a-b** were performed as described previously.[1] Product standard (*R*/*S*)-levodione **6c** was synthesised as described previously.[2] All monoterpenoid compounds were dissolved as stock solutions in absolute ethanol, with a final concentration of 2 % (v/v) in the reactions. The concentration of nicotinamide coenzymes (Melford) was determined by the extinction coefficient method (ε_{340} = 6220 $M^{-1}cm^{-1}$). Steady-state kinetic analyses were performed on a Cary UV-50 Bio UV/Vis scanning spectrophotometer using a quartz cuvette (1 mL; Hellma) with a 1 cm path length. Reaction extracts (1 □L) were analysed by GC and/or GCMS using an Agilent Technologies 7890A GC system with FID detector. Analyses were performed on a DB-WAX column (30 m; 0.32 mm; 0.25 μm film thickness; JW Scientific) using the monoterpenoid separation method described previously.[3] Unknown products were analysed on an Agilent Technologies 7890B GC system with a 5977A MSD extractor EI source detector using the same DB-WAX column. In this method the injector temperature was at 240°C with a split ratio of 50:1 (1 □L injection). The carrier gas was helium with a flow rate of 3 $mLmin^{-1}$ and a pressure of 8.3 psi. The program began at 40°C with a hold for 2 min followed by an increase of temperature to 210°C at a rate of 15°C/minute, with a hold at 210 °C for 3 min. The mass spectra fragmentation patterns were entered into the NIST/EPA/NIH 11 (mass spectral library for identification of a potential match. Product enantiomeric excess was determined by analysing reactions by GC using a Chirasil-DEX-CB column (Varian; 25 m, 0.32 mm, 0.25 μm). In this method the injector temperature was at 180°C with a split-less injection. The carrier gas was helium with a flow rate of 1 $mLmin^{-1}$ and a pressure of 5.8 psi. The program began at 70°C with an increase of temperature to 150°C at a rate of 20°C/minute and a hold for 3 min. This was followed by an increase of temperature to 190°C at a rate of 2°C/minute and a hold for 3 min.

Synthesis of verbanone 6d

Verbenone (0.5 g, 3.3 mmol) was added to MeOH (10 mL) followed by the addition of Pd/C (50 mg, 15 mol %). Hydrogen gas was passed over solution for 2 hours and the reaction was filtered and the MeOH dried and removed under vacuum. This gave a white solid (0.4 g, 2.8 mmol, 85 % yield). ^{1}H NMR (400 MHz, $CDCl_3$) δ 2.86 (1H, dd, *J* 20 Hz, 10 Hz), 2.52-2.61 (2H, m), 2.36-2.40 (1H, m, H_D), 2.15 (1H, dd, *J* 20 Hz, *J* 4 Hz, H_E), 1.4 (1H, d, *J* 12 Hz), 1.33 (3H, s, $CH3_B$), 1.15 (

3H, d, *J* 8 Hz, $CH3_C$), 1.0 (3H, s, $CH3_A$). ^{13}C NMR (100 MHz, $CDCl_3$) δ 214 (C=O), 58.0, 47.4, 41.4, 40.3, 31.1, 28.5, 27.0, 24.5, 21.1. Data match those previously reported.[4]

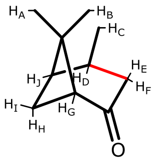

Synthesis of 3,5-dimethylcyclohex-2-en-1-ol 7

(±)-3,5-Dimethyl-1-cyclohexen-1-one (0.568 g, 4.6 mmol), sodium borohydride (0.338 g, 9.16 mmol, 2 eq) were added to MeOH (10 mL) and left to stir at room temperature for 3 hours. The reaction was quenched with H_2O (20 mL) and the mixture was extracted with ethyl acetate (3 x 25 mL), dried with $MgSO_4$ and concentrated under reduced pressure. This gave the title compound as a yellow oil (0.4 g, 3.2 mmol, 70 % yield). ^{1}H NMR (400 MHz, $CDCl_3$) δ 5.38-5.36 (1H, m, H_B), 4.26-4.25 (1H, m, H_A), 2.02-1.98 (1H, m, H_G), 1.91-1.90 (2H, m, H_I+H_H), 1.65 (3H, s, $CH3_C$), 1.1 (1H, d, *J* 8 Hz, H_E), 0.99 (3H, d, *J* 8 Hz, $CH3_F$), 0.9 (1H, d, *J* 8 Hz, H_D). ^{13}C NMR (100 MHz, $CDCl_3$) δ 136.8 (C-OH), 125.5, 68.5, 41.4, 38.9, 28.2, 23.1, 21.9. Minor diastereoisomer peaks are visible.

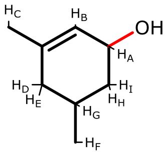

Enzyme production and purification

The protein sequences for the following *M. piperita* enzymes were obtained from UniProt (http://www.uniprot. org): *i*) MMR (Q5CAF4), *ii*) MNMR (Q06ZW2) and *iii*) IPR (Q6WAU1). The respective gene sequences were designed and synthesised by GenScript (USA), incorporating codon optimisation techniques of rare codon removal for optimal expression in *E. coli*. The genes for MMR and IPR[1] were sub cloned individually into pET21b (Novagen) via *Nde*I/*Xho*I restriction sites, without a stop codon, to incorporate a C-terminal His_6-tag. In the case of MNMR, a highly expressing construct was produced by sub cloning the gene into pET28b, via *Nde*I/*Xho*I restriction sites, to generate a N- and C-terminally His_6-tagged protein for characterisation studies. Each construct was transformed into the *E. coli* strain BL21(DE3)pLysS (Stratagene) for soluble protein over-expression according to the manufacturer's protocol. A general His_6-tagged protocol for the production and purification of each individual His_6-tagged protein was performed as described previously.[1, 3] In the case of IPR, an additional purification step was performed using a Q Sepharose column (GE Healthcare) pre-equilibrated in buffer A (50 mM Tris pH 8.0 containing 1 mM β-mercaptoethanol and 10% glycerol). Purified IPR was eluted in a gradient to buffer B (50 mM Tris pH 8.0 containing 1 M NaCl, 1 mM β-mercaptoethanol and 10% glycerol). All purified enzymes were dialysed into cryobuffer (10 mM Tris pH 7.0 containing 10 % glycerol), and flash frozen in liquid nitrogen for storage at -80°C. Protein concentration was determined using the Bradford and extinction coefficient methods.[5] Purity was assessed by SDS-PAGE, using 10-12% Mini-PROTEAN® TGX Stain-Free™ gels and Precision Plus protein unstained markers (BioRad) according to the manufacturers instructions.

Additional IPR and MNMR constructs were generated in pETM11 containing only a cleavable N-terminally His_6-tagged for protein crystallisation studies. The genes encoding IPR and MNMR were amplified from the pET21b constructs, using primer sets TTTCAGGGCGCCATGGC GGAAGTCCAACGCTATGCTC/ GGTGGTGGTGCTCGATTAATACAGAGCCAGTGCTTTGT CACG and TTTCAGGGCGCCATGGGTGACGAAGTGGTTGTGGATC/GGTGGTGGTGC TCGATCAATACAGACAAAACGCTTCATCG respectively. A modified pET24b vector (pET-M11) was digested with restriction enzymes NcoI and XhoI. The amplified genes were cloned into pET-M11 vector using the InFusion cloning (Clontech) technique, according to the manufacturers protocol. The final constructs contain a N-His_6 tag followed by a TEV protease cleavage site.

The constructs were transformed into BL21 (DE3) cells according to the manufacturers instructions. Starter cultures were composed of terrific broth (50 mL) containing kanamycin (15 μgmL^{-1}) and thr *E. coli* constructs, and incubated at 37°C for 4 hours. This was diluted into further terrific broth medium (2 L) containing kanamycin (15 μgmL^{-1}) and was grown at 37°C until the OD_{600} reached 0.5-0.7. The temperature was reduced to 18°C and recombinant protein expression was induced by the addition of IPTG (0.1 mM). After 15 hours, cells were harvested by centrifuging at 6000*g* for 10 minutes. The cells were resuspended in lysis buffer (25 mM Tris-HCl pH 8.0) containing 150 mM NaCl, 1X protease inhibitor cocktail (Roche) and DNase (10 µg/ml). The cells were lysed using a sonicator (Bandelin) with a probe set at 40% amplitude with cycles of 10 seconds ON and 20 seconds OFF for 10 minutes. The lysed cells were centrifuged at 40000g for 30 minutes and the supernatant was passed through a 0.2 micron filter and injected onto a 5ml HisTrap column (GE HealthCare). The column was washed with lysis buffer containing up to 40mM imidazole. The enzymes were eluted with a gradient from 40 to 200 mm imidazole.

The proteins were passed through a gravity-flow desalting column (CentriPure P100) equilibrated with lysis buffer and treated with TEV protease at 4°C for 16 hours. To remove the His-tagged TEV protease, samples were loaded onto a 5ml HisTrap column. The flow through from the HisTrap column containing IPR or MNMR without the His tag was concentrated using the VivaSpin centrifugal concentrator (10kDa cutoff) and injected onto a Hiload Superdex 75 26/60 column. The pure fractions from the gel filtration chromatogram peak were concentrated to 15 mg/ml.

Site directed mutagenesis of MNMR and IPR. Variants of MNMR (Y244E) and IPR (E238Y) were generated by site-directed mutagenesis using the Stratagene QuickChange whole plasmid synthesis protocol. The PCR reaction with IPR was performed using the C-terminally His_6-tagged gene in pET21b and the following oligonucleotides: CCGCATTTCGCAGCT*TAC*CG TGTGTCAAAGGCG and CGCCTTTGACACACG*GTA*AGCTGCGAAATGCGG. For MNMR mutagenesis, both the constructs in pET28b and pETM11 were used with the following oligonucleotides: GCCGCATTTCAGTGCT*GAA*AAAGTCTCCAAGGCGG and CCGCCTTGG AGACTTT*TTC*AGCACTGAAATGCGGC. Base substitutions in the oligos are shown in italics. Following template removal by selective restriction digest (DpnI), PCR products (~50 ng) were transformed into the *E. coli* strain BL21(DE3)pLysS according to the manufacturer’s protocol. Each mutant was grown on LB agar containing ampicillin (100 μgmL^{-1}; IPR) or kanamycin (34 μgmL^{-1}; MNMR) for 24 h at 37°C. Colonies (3) of each mutant were grown and fully sequenced to confirm the presence of the required mutation. Variant enzyme production and purification was performed as for the wild-type enzymes, except no Q-Sepharose step was performed for MNMR.

Enzyme kinetics. Standard ketoreductase reactions (1 mL) were performed in KR buffer (50 mM Tris pH 7.0) containing dithiothreitol (1 mM; DTT), NADPH (50 μM), monoterpenoid (1 mM) and enzyme (30 nM to 2 μM). Reactions were followed by continuously monitoring NADPH oxidation at 340 nm for 1 min at 25°C. Standard double bond reductase reactions (1 mL) were performed in DB buffer (50 mM KH_2PO_4, 12.5 mM K_2HPO_4 pH 5.5-5.8 for IPR and 50 mM Tris pH 7.0 for MMR/MNMR) containing dithiothreitol (1 mM; DTT), NADPH (100 μM), monoterpenoid (1 mM) and enzyme (30 nM to 2 μM). Reactions were monitored continuously at 340 nm as described above. To determine the optimal pH for each enzyme in both steady state and biotransformations, reactions were performed in a buffer composed of three buffer salts (12.5 mM tri-sodium citrate, 12.5 mM KH_2PO_4, 12.5 mM K_2HPO_4 and 12.5 mM CHES) instead of using Tris buffer. All steady state reactions were performed in at least duplicate.

Biotransformations. Ketoreductase reactions (1.0 mL) with purified enzymes were performed in BT buffer (50 mM Tris pH 7.0) containing the monoterpenoid (5 mM), $NADP^+$ (10 μM), glucose (15 mM), glucose dehydrogenase (GDH from *Pseudomonas* sp.; 10 U; Sigma Aldrich) and enzyme (2-5 μM). Reactions were shaken at 30°C for 24 h at 130 rpm, and terminated by extraction with ethyl acetate (0.9 mL) containing an internal standard (0.05 % (*S*)-limonene or 0.1 % *sec*-butylbenzene). All reaction extracts were dried using anhydrous magnesium sulphate, and analysed by GC. Double bond reductase reactions were performed in a similar manner, except the buffer was composed of phosphate buffer (50 mM KH_2PO_4/K_2HPO_4 pH 5.5) for IPR and BT buffer (50 mM Tris pH 7.0) for MMR/MNMR. Quantitative analysis was carried out by a comparison of product peak areas to standards of known concentrations. Products were identified by comparison with authentic standards. All biotransformation reactions were performed in at least duplicates, and the results are averages of the data.

Protein crystallography

Crystallisation trials were setup using a Mosquito robot (TTP Labtech) with a drop ratio of 1:1 in MRC 3-drop plates and incubated at 4°C. Eight commercially available screens (Molecular Dimensions and Microlytic) were used for initial screening. IPR crystals were obtained in several conditions in the Morpheus screen.[6] The X-ray data for IPR were collected from crystals grown in well positions C1, D1, E1, F1, and G1. The MNMR crystals were obtained when 200 nL of protein was mixed with 200 nL of mother liquor containing 0.1M imidazole pH 8 and 1M sodium citrate. Before flash cooling crystals with liquid nitrogen, IPR crystals were cryo-protected by washing with mother liquor, while MNMR crystals were cryo-protected by washing with mother liquor supplemented with 20% glycerol. For obtaining protein-ligand complexes, IPR crystals were incubated for 1 hour in mother liquor supplemented with 10mM NADPH and either 25 mM **3a** or 25 mM β-cyclocitral. Similarly, MNMR crystals were incubated in mother liquor supplemented with 10 mM NADPH and 20% glycerol.

Structure solution for IPR and MNMR

The X-ray data were collected at Diamond Light Source beamlines I02, I03 and I04. MNMR datasets and IPR bound to β-cyclocitral were processed by automated pipeline implemented in xia2,[7] using xds[8] and xscale. Other IPR datasets were processed manually using DIALS and aimless[9] in the CCP4 suite.[10] The IPR structure bound to $NADP^+$ was solved by molecular replacement using SalR structure (PDB 3O26) as the search model in Phaser.[11] The structures of IPR bound to **3a** or β-cyclocitral were subsequently solved using difference Fourier methods. The

MNMR structures were solved by molecular replacement using the IPR structure as the search model. Automated model building by Autobuild,[12] as implemented in Phenix,[13] built the majority of the residues for both IPR and MNMR. The structures were completed by repeated rounds of manual model building in Coot[14] and refinement using phenix_refine.[15] The structures were validated using Molprobity[16] and PDB_REDO.[17] The crystallograpic data summary and refinement parameters are listed in Table S2. The atomic coordinates and structure factors (codes 5LCX: IPR/NADP; 5LDG: IPR/NADP/**3a**; 5L4S: IPR/NADP/β-cyclocitral; 5L51: apo-MNMR and 5L53: MNMR/NADP) have been deposited in the Protein Data Bank, Research Collaboratory for Structural Bioinformatics, Rutgers University, New Brunswick, NJ (http://www.rcsb.org/).

2. Supplementary enzyme crystal structure discussion

The unique difference between the structures exists in the "flap-like" domain: SalR's flap domain is composed of 2 short β-strands connected by 3 α-helices whereas the IPR's flap domain is composed of 2 β-strands connected by a single elongated α-helix and a long loop (Figure 1a). Importantly, the IPR's flap domain is twisted inwards, compared to the SalR's flap domain, which aligns the α-helix almost parallel to the β-sheet of the core domain. Surface representation of SalR structure shows it is exposed to the solvent from the nicotinamide end of $NADP^+$, but the rotation of the flap domain in IPR closes this solvent channel (Figure S7). IPR also shares structural similarities with carbonyl reductases (CBRs) with the core structures being homologous but differ in the region that caps the substrate. In human CBR[18], there is no flap domain and instead a short loop connects α4 and β4 and leaves the substrate-binding region open at the top (Figure S8a). In chicken liver CBR[19] a different loop, which connects α5 and β5, extends long and acts as a cap for the substrate (Figure S8b). Analysis of IPR homologs (396 sequences) using the ConSurf server[20] revealed the presence of highly conserved residues around the $NADP^+$ binding site and most of the residues in the flap domain are highly variable (Figure S9). Based on the above factors, it is clear that the flap domain in IPR/SalR is species specific. Moreover, it is unlikely to interact with the substrate directly (see below) and might be involved in interaction with partner protein(s) in their respective pathways.

3. Supplementary Figures

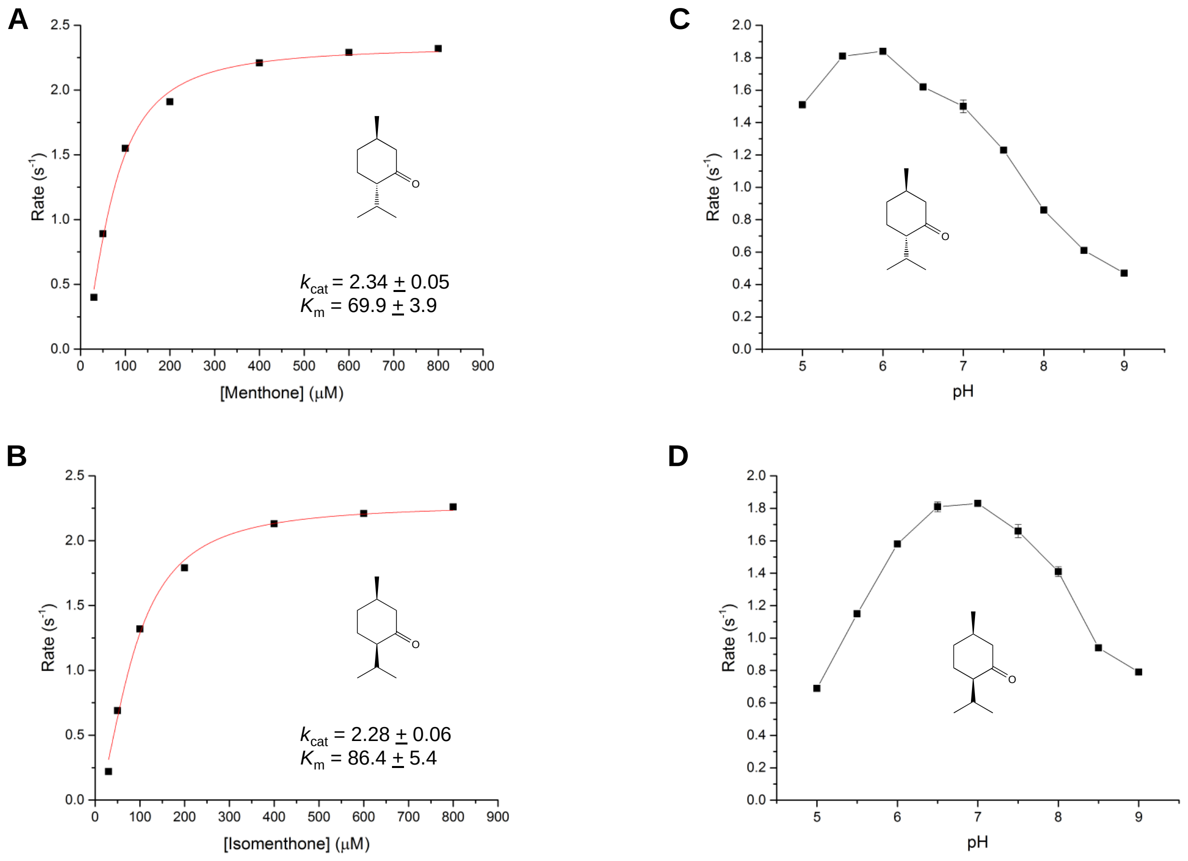

Figure S1. Steady state kinetics of MMR with substrates **1a** and **1b**. Michaelis Menten plot of MMR with A) **1a** and B) **1b**. Reactions (1 mL) were performed in buffer (50 mM Tris pH 7.0) containing dithiothreitol (1 mM), NADPH (50 µM), monoterpenoid (1 mM) and enzyme (30 nM to 2 µM). Reactions were followed by continuously monitoring NADPH oxidation at 340 nm for 1 min at 25°C. Optimal pH determination of MMR with C) **1a** and D) **1b**. Reactions were performed as above, except the reaction buffer differed (12.5 mM tri-sodium citrate, 12.5 mM KH_2PO_4, 12.5 mM K_2HPO_4 and 12.5 mM CHES).

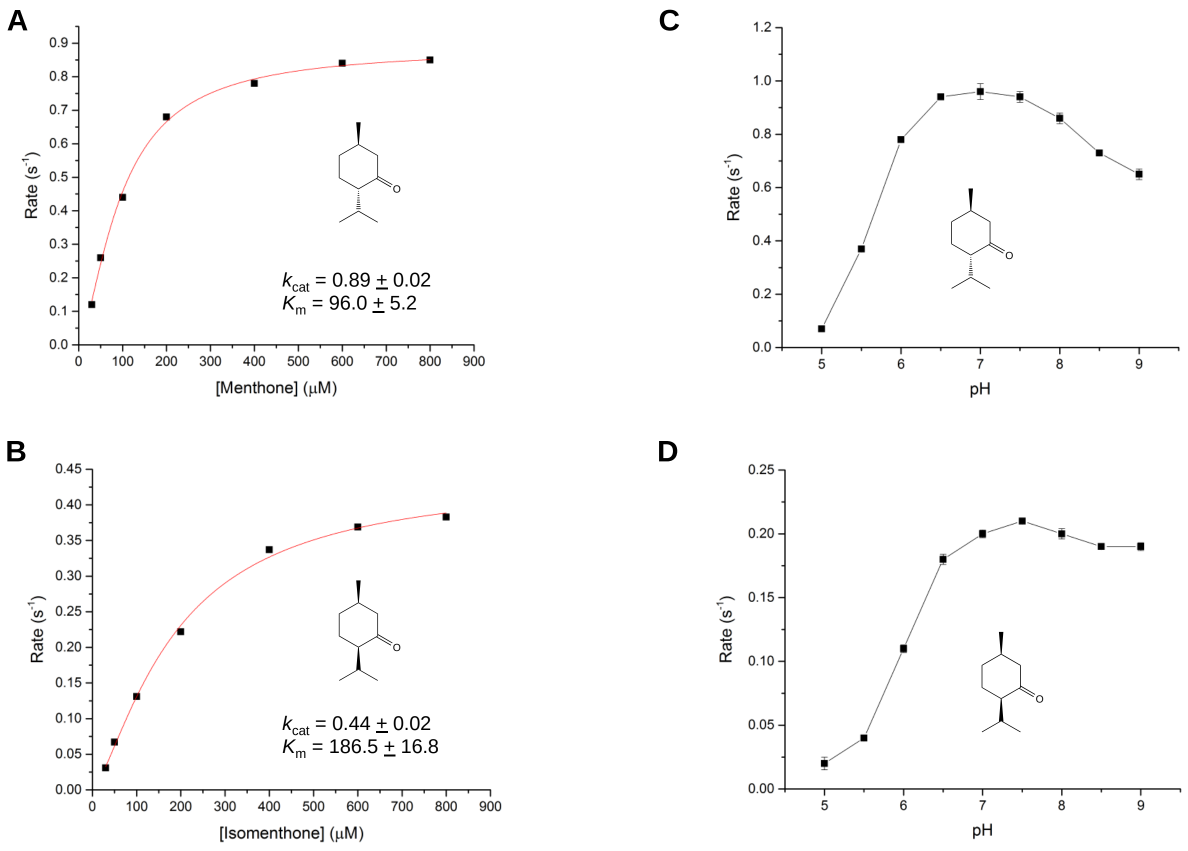

Figure S2. Steady state kinetics of MNMR with substrates **1a** and **1b**. Michaelis Menten plot of MNMR with A) **1a** and B) **1b**. Reactions (1 mL) were performed in buffer (50 mM Tris pH 7.0) containing dithiothreitol (1 mM), NADPH (50 µM), monoterpenoid (1 mM) and enzyme (30 nM to 2 µM). Reactions were followed by continuously monitoring NADPH oxidation at 340 nm for 1 min at 25°C. Optimal pH determination of MNMR with C) **1a** and D) **1b**. Reactions were performed as above, except the reaction buffer differed (12.5 mM tri-sodium citrate, 12.5 mM KH_2PO_4, 12.5 mM K_2HPO_4 and 12.5 mM CHES).

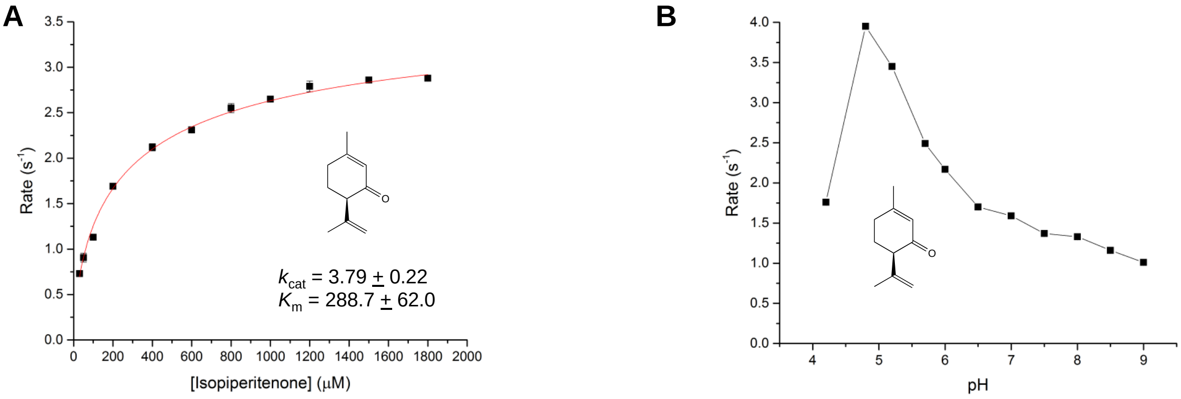

Figure S3. Steady state kinetics of IPR with **3a**. A) Michaelis Menten plot of IPR with **3a**. Reactions (1 mL) were performed in buffer (50 mM KH_2PO_4, 12.5 mM K_2HPO_4 pH 5.5) containing dithiothreitol (1 mM), NADPH (50 μM), monoterpenoid (1 mM) and enzyme (30 nM to 2 μM). Reactions were followed by continuously monitoring NADPH oxidation at 340 nm for 1 min at 25°C. B) Optimal pH determination of IPR with **3a**. Reactions were performed as above, except the reaction buffer differed (12.5 mM tri-sodium citrate, 12.5 mM KH_2PO_4, 12.5 mM K_2HPO_4 and 12.5 mM CHES).

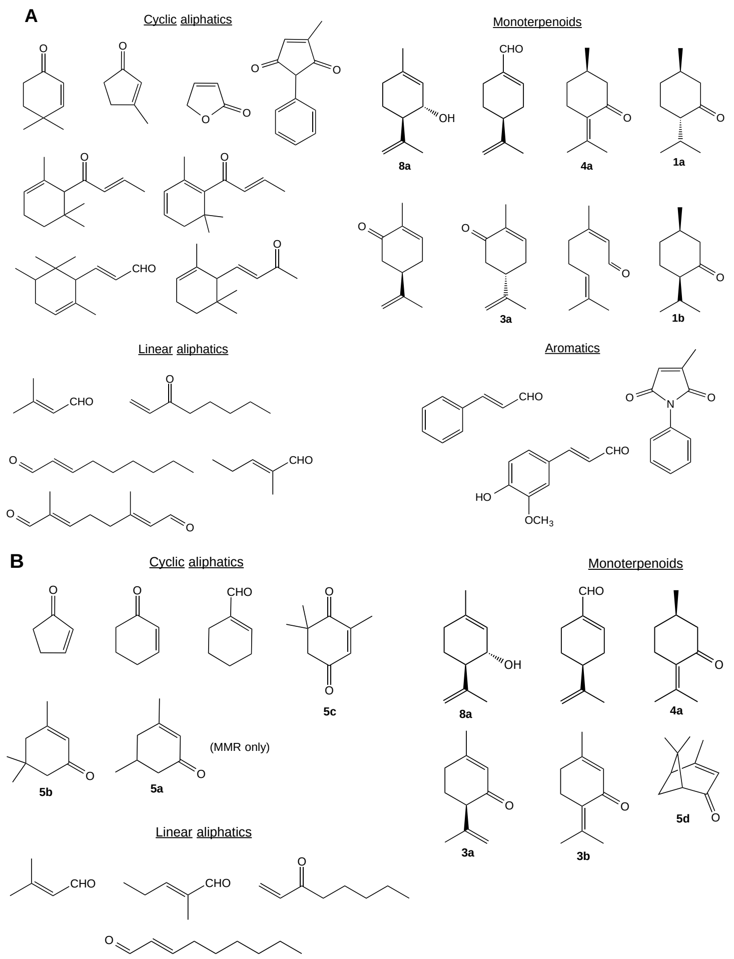

Figure S4. α,β-Unsaturated enones, enals and enols not reduced by wild type enzymes A) IPR or B) MMR and MNMR.

```
                            TGxxxGhG
SALR      -MPETCPNTVTKRRCAVVTGGNKGIGFEICKQLSSNGIMVVLTCRDVTKGHEAVEKLKN-
MNMR      MGDEVVVDHAATKRYAVVTGANKGIGFEICKQLASKGITVILASRDEKRGIEARERLIKE
MMR       ------MADTFTQRYALVTGANKGIGFEICRQLASKGMKVILASRNEKRGIEARERLLKE
......................................................................
IPR       --------MAEVQRYALVTGANKGIGFEICRQLAEKGIIVILTSRNEKRGLEARQKLLKE
                   .  :* *:***.*********:**.:*: *:*:.*: .:* ** ::* :
                        D                   GxhDhhhNNAGh
SALR      ---SNHENVVFHQLDVTDPIATMSSLADFIKTHFGKLDILVNNAGVAGFSVDADRF--KA
MNMR      LGSEFGDYVVSQQLDVADPAS-VAALVDFIKTKFGSLDILVNNAGLNGTYMEGDASVLND
MMR       SRSISDDDVVFHQLDVADPAS-AVAVAHFIETKFGRLDILVNNAGFTGVAIEGDISVYQE
......................................................................
IPR       LNV-SENRLVFHQLDVTDLAS-VAAVAVFIKSKFGKLDILVNNAGVSGVEMVGDVSVFNE
                : :* :****:*  :   ::. **:::** *********. *  : .*    :

SALR      MIS----DIG-EDSEELVKIYEKPEAQELMSETYELAEECLKINYNGVKSVTEVLIPLLQ
MNMR      YVEAEFKTFQ---SGAAKTEPYHPKATGRLVETVEHAKECIETNYYGSKRVTEALIPLLQ
MMR       CLEANIIAAQ---GG--QAHPFHPKTTGRLIETLEGSKECIETNYYGTKRITETLIPLLQ
......................................................................
IPR       YIEADFKALQALEAGAKEEPPFKPKANGEMIEKFEGAKDCVVTNYYGPKRLTQALIPLLQ
           :.          .        :*::   : *. * :::*:  ** * * :*:.******
                     S
SALR      LSDSPRIVNVSSSTGSLKYVSNETALEILGDGDALTEERIDMVVNMLLKDFKENLIETNG
MNMR      QSDSPRIVNVSSTLSSLVFQTNEWAKGVFSSEEGLTEEKLEEVLAEFLKDFIDGKQQEKQ
MMR       KSDSPTIVNVSSTFSTLLLQPNEWAKGVFSSN-SLNEGKVEEVLHEFLKDFIDGKLQQNH
......................................................................
IPR       LSPSPRIVNVSSSFGSLLLLWNEWAKGVLGDEDRLTEERVDEVVEVFLKDIKEGKLEESQ
           * ** ******: .:*    ** *  ::..   *.* ::: *:  :***: :   : .
                 Y   K                            T
SALR      WPSFGAAYTTSKACLNAYTRVLANKIPKFQVNCVCPGLVKTEMNYGIGNYTAEEGAEHVV
MNMR      WPPHFSAYKVSKAALNAYTRIIAKKYPSFRINAVCPGYTKTDLSYGHGQFTDAEAAEAPV
MMR       WPPNFAAYKVSKAAVNAYTRIIARKYPSFCINSVCPGFVRTDICYNLGVLSEAEGAEAPV
......................................................................
IPR       WPPHFAAERVSKAALNAYTKIAAKKYPSFRINAICPGYAKTDITFHAGPLSVAEAAQVPV
          **   :*  .***.:****:: *.* *.* :*.:*** .:*:: :  *  :  *.*:  *

SALR      RIALFPDDGPSGFFYDCSELSAF-
MNMR      KLALLPQGGPSGCFFFRDEAFCLY
MMR       KLALLPDGGPSGSFFSREEALSLY
..................................
IPR       KLALLPDGGPSGCFFPRDKALALY
          ::**:*: **** *:  .:  .:
```

Figure S5. Primary sequence alignment of four SDR enzymes. The three ketoreductases are salutaridine reductase (SALR; UniProt ID: Q071N0) from *Papaver somniferum* L,[21] and two *M. piperita* enzymes menthone:(+)-neomenthol reductase (MNMR; UniProt ID: Q06ZW2) and (−)-menthone:(−)menthol reductase (MMR; UniProt ID: Q5CAF4).[22] The double bond reductase IPR (isopiperitenone reductase from *M. piperita;* UniProt ID: Q6WAU1)[23] is separated from the ketoreductases by a dotted line. Conserved residue positions highlighted in blue and green are involved in the binding of the nicotinamide coenzyme and stabilisation of the central β-sheet, respectively. Active site residues are shown in red. The sequence alignment was performed by Clustal Omega (http://www.ebi.ac.uk/Tools/msa/clustalo/).

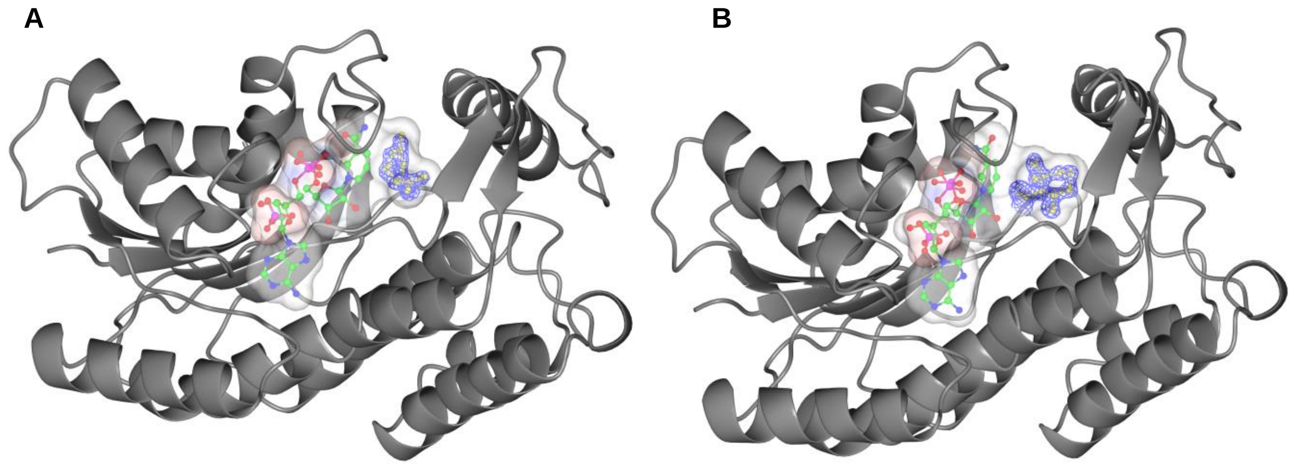

Figure S6. IPR structures containing bound (A) 3a and (B) β-cyclocitral. The substrates are coloured in yellow, and $NADP^+$ molecules are shown as atom coloured sticks with yellow carbons. The *fo-fc* densities (blue mesh), contoured at 3σ, are shown for the substrates. This figure was prepared using CCP4mg[24].

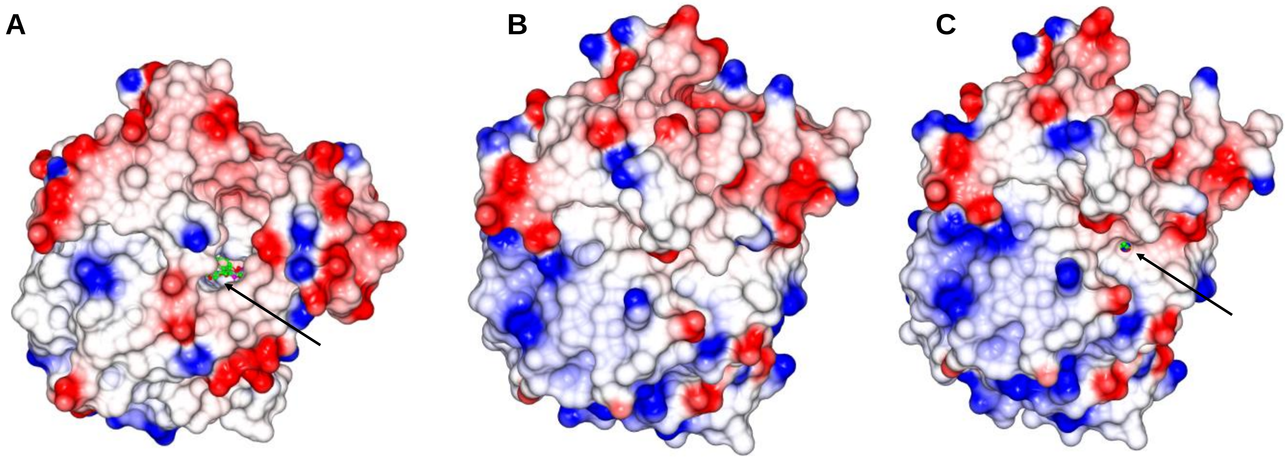

Figure S7. Surface representation of SalR and IPR structures. Side views of (A) SalR (PDB 3O26), (B) IPR bound to $NADP^+$ and (C) IPR bound to $NADP^+$ and **3a**. Solvent exposed nicotinamide ring of $NADP^+$ in SalR and **3a**-bound IPR structures are indicated by arrows. This figure was prepared using CCP4mg[24].

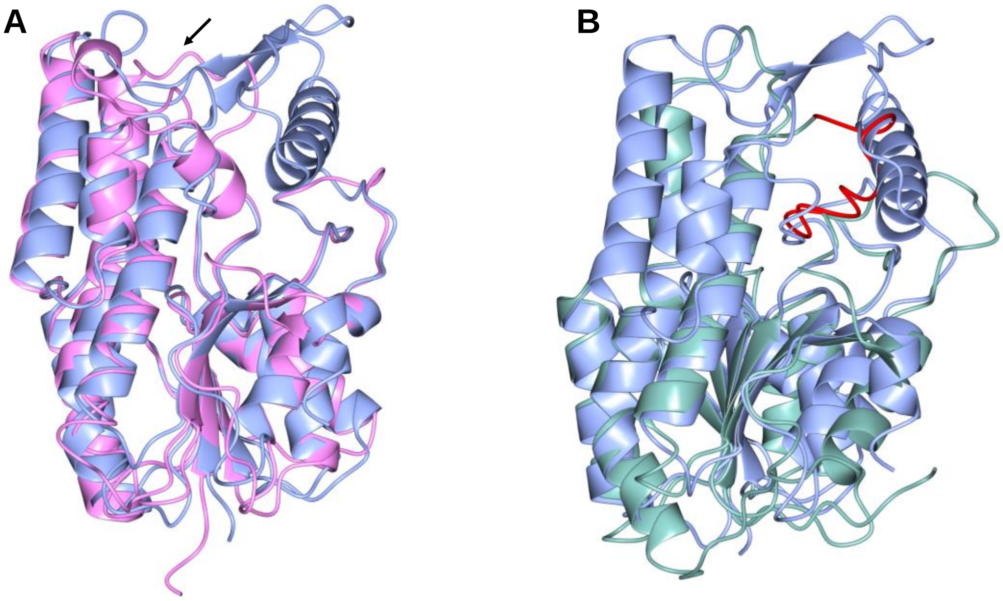

Figure S8. Structural comparison of IPR with carbonyl reductases. **(**A) Overlay of IPR (blue) with human carbonyl reductase1 (CBR1, pink) (PDB 1WMA). The loop in CBR1 which connects α4 and β4 (in the place of flap domain in IPR), is indicated by an arrow. (B) Overlay of IPR with chicken liver carbonyl reductase (green) (PDB 3WXB). The long loop, which connects α5 and β5 and caps the active site in chicken carbonyl reductase is shown in red. This figure was prepared using CCP4mg[24].

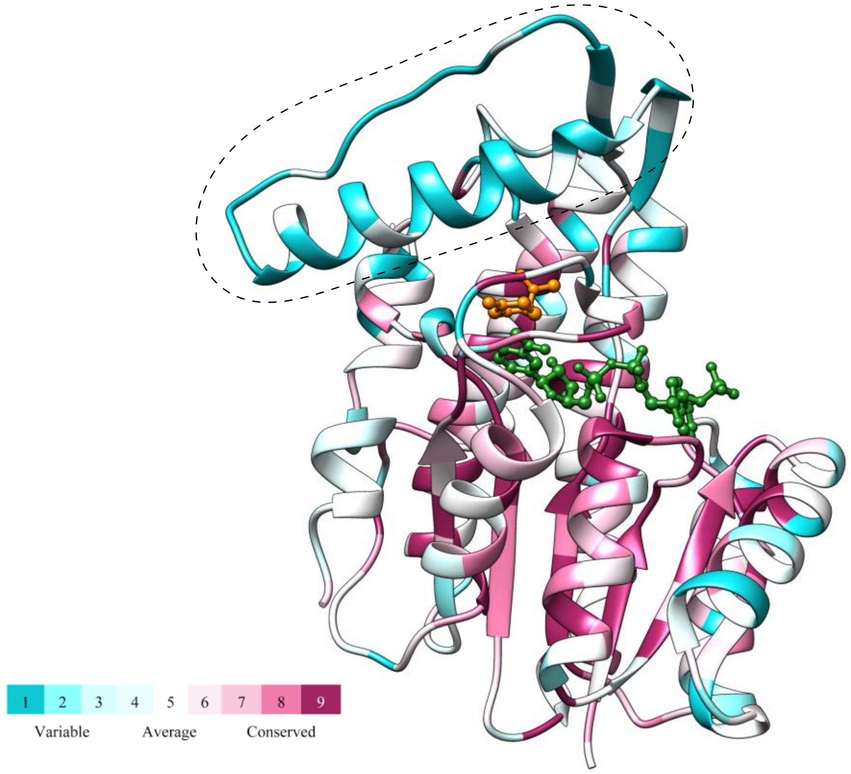

Figure S9. Conserved surface analysis of IPR. ConSurf server[20] was used to map the conserved residues based on 369 IPR homologous sequences. NADP$^+$ and **3a** are displayed as ball and stick and are coloured green and orange respectively. The variable flap domain is indicated by dotted lines. This figure was prepared using Chimera[25].

A)

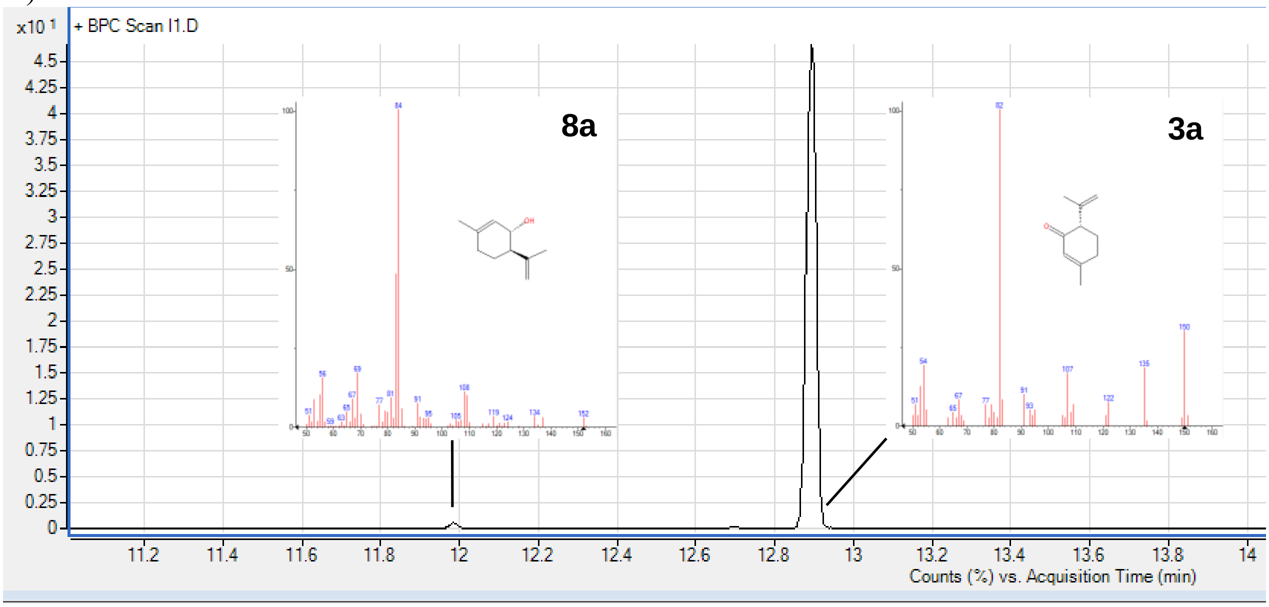
x10 1
+ BPC Scan I1.D
8a
3a
Counts (%) vs. Acquisition Time (min)

B)

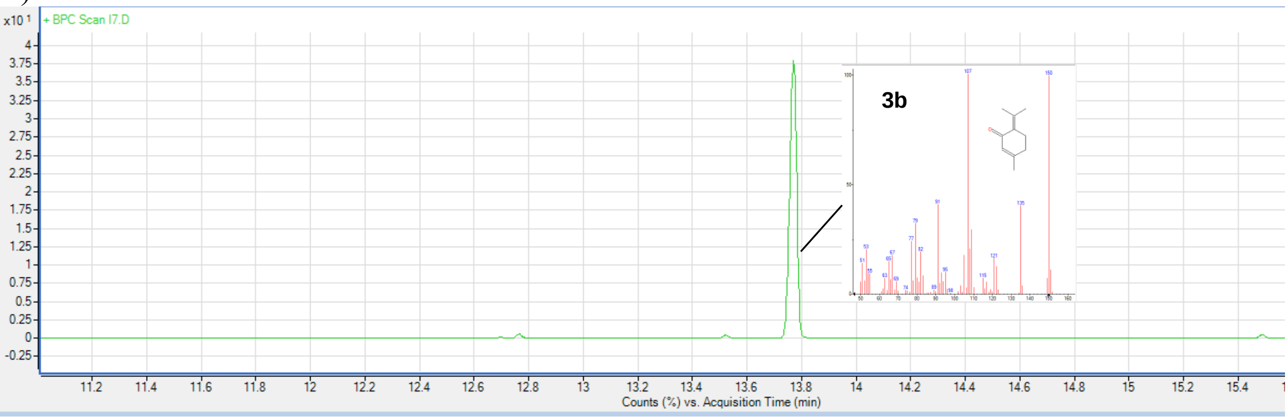
x10 1
+ BPC Scan I7.D
3b
Counts (%) vs. Acquisition Time (min)

C)

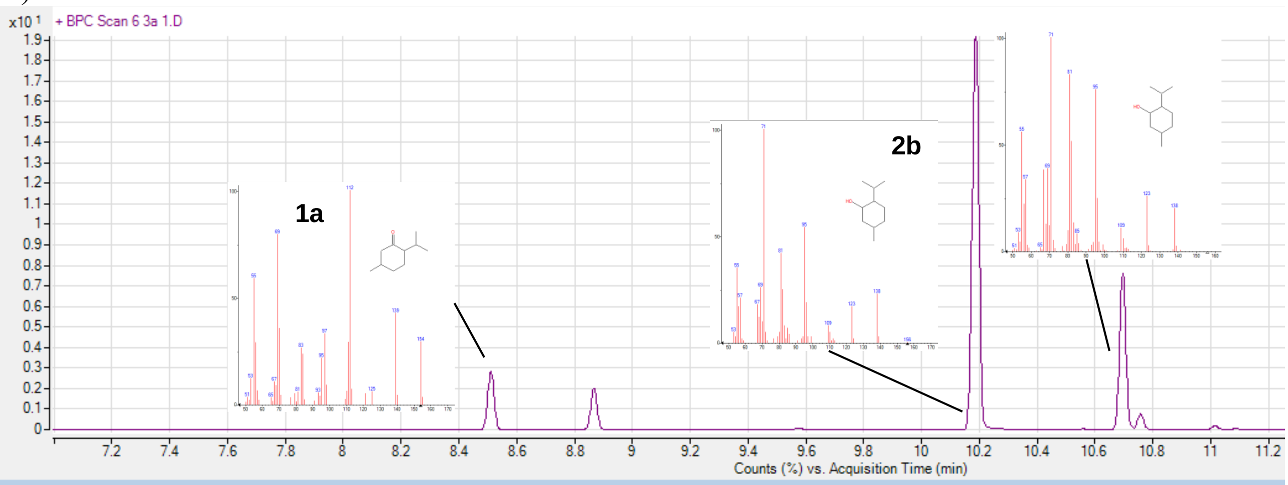
x10 1
+ BPC Scan 6 3a 1.D
1a
2b
Counts (%) vs. Acquisition Time (min)

D)

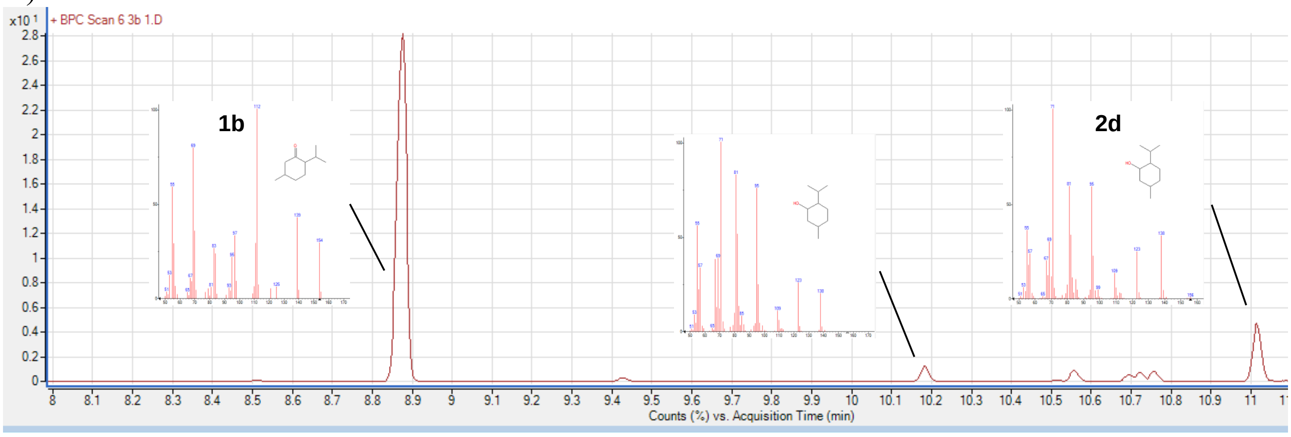

E)

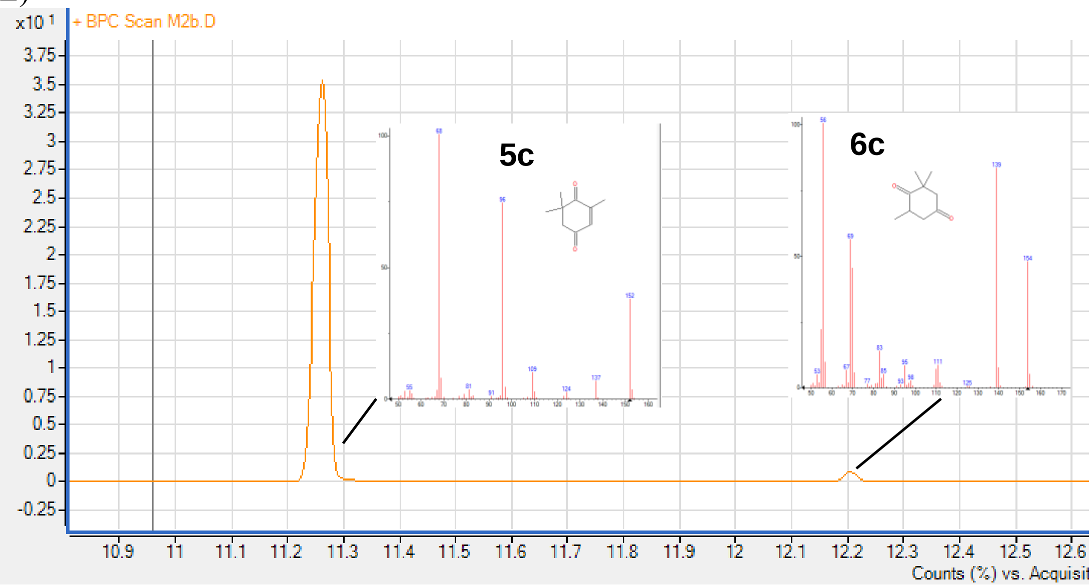

Figure S10. GCMS chromatograms and MS identification of the products of variant enzyme reactions: A) IPR E238Y with **3a**; B) IPR E238Y with **3b**; C) IPR E238Y with **1a**; D) IPR E238Y with **1b**; E) MNMR Y244E with **5c**.

4. NMR Spectra

Figure S6. ^{1}H and ^{13}C NMR spectra for synthesised compounds.

Verbenone, 6d

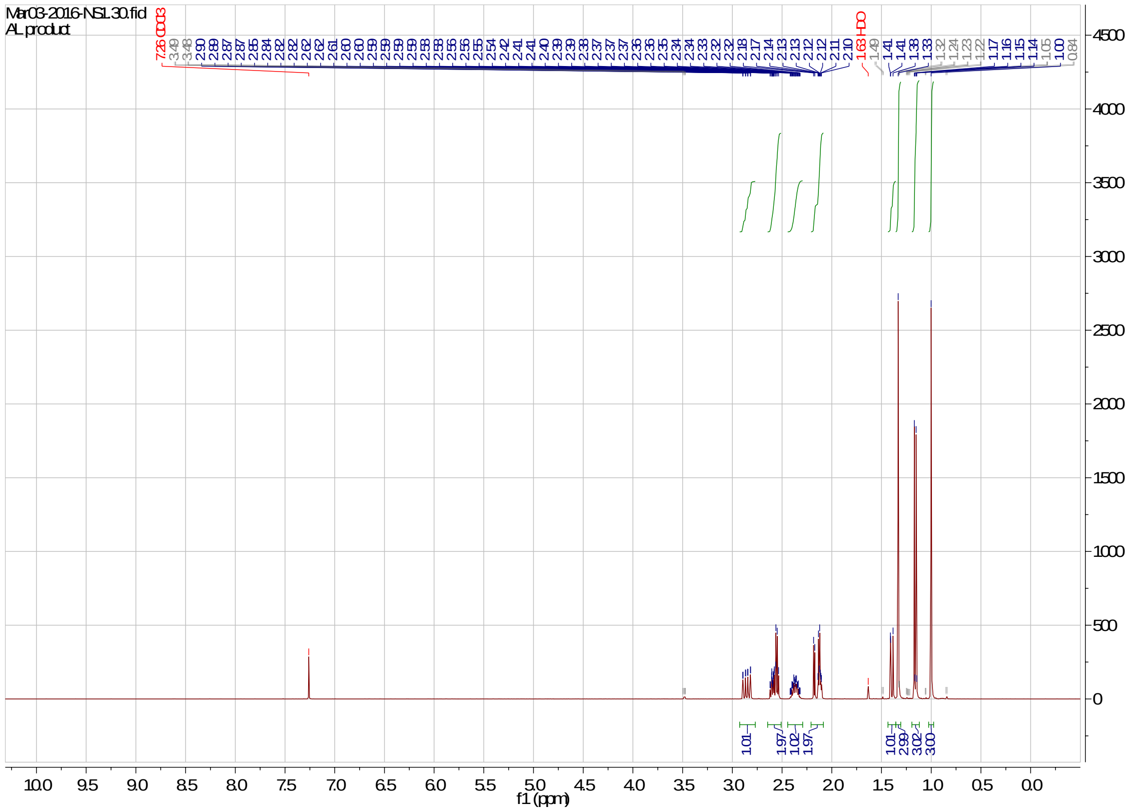

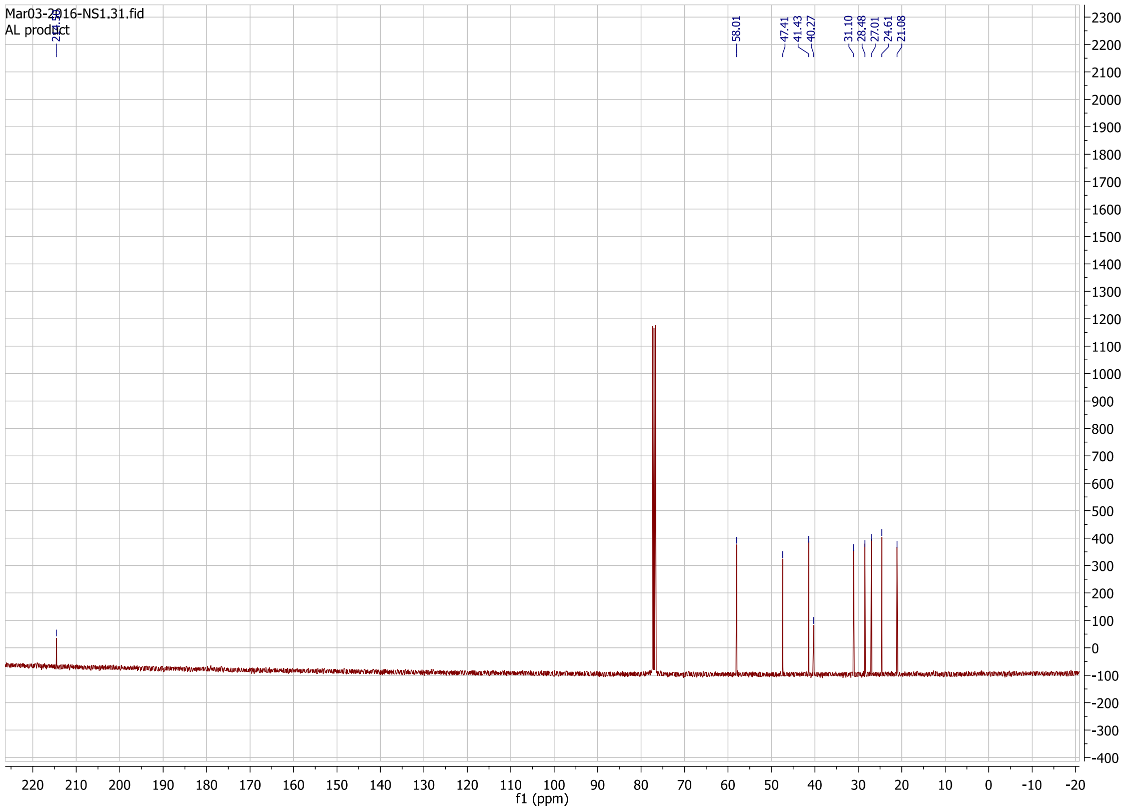

3,5-dimethylcyclohex-2-en-1-ol, 7

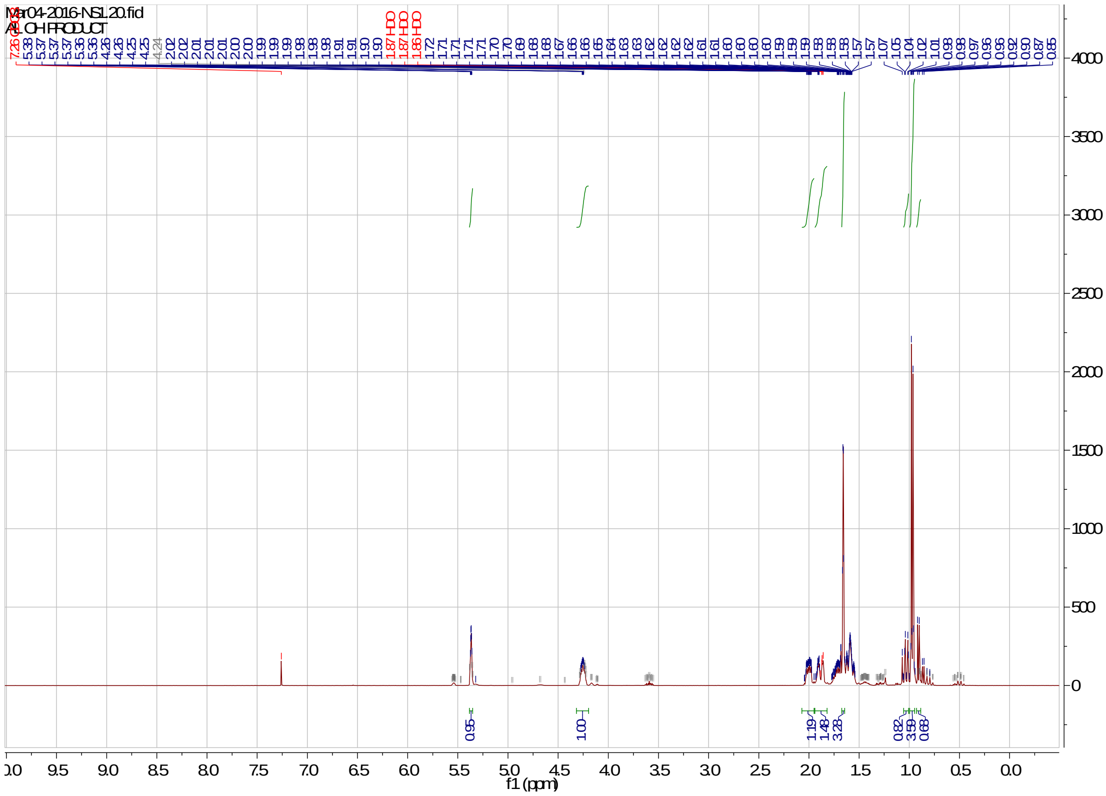

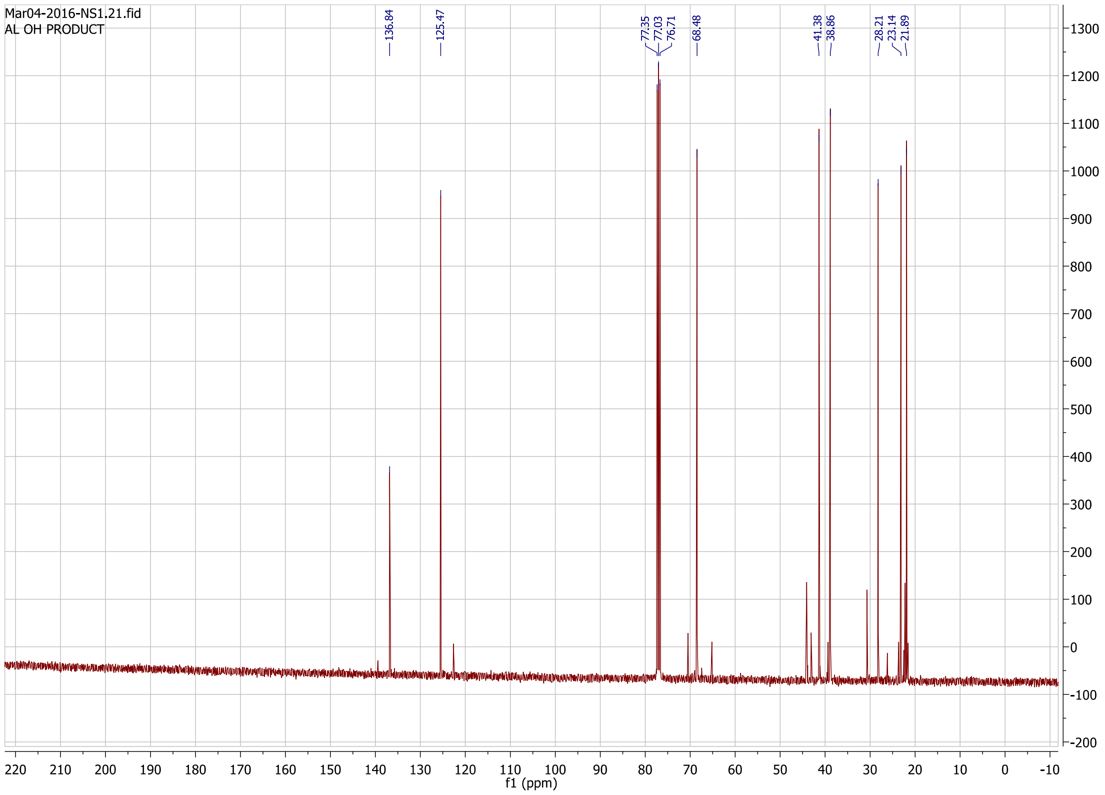

5. Supplementary Tables

Table S1. pH profiles of IPR, MMR and MNMR.

Enzyme	Substrate	pH Optimum
IPR	**3a**	5.0
MMR	**1a**	6.0
	**1b**	7.0
MNMR	**1a**	6.5-7.5
	**1b**	7.5

Reactions (1 mL) were performed in buffer (12.5 mM tri-sodium citrate, 12.5 mM KH_2PO_4, 12.5 mM K_2HPO_4 and 12.5 mM CHES) containing monoterpenoid (1 mM), enzyme (30 nM to 2 μM), dithiothreitol (1 mM; DTT) and NADPH (100 μM). Reactions were followed by continuously monitoring NADPH oxidation at 340 nm for 1 min at 25°C.

**Table S2.** X-ray data collection and refinement statistics.					
	IPR-NADP$^+$	IPR (NADP$^+$/ Isopiperitenone)	IPR (NADP$^+$/ β-cyclocitral)	MNMR-Apo	MNMR-NADP$^+$
**Data collection**					
Space group	$P2_12_12_1$	$P2_12_12_1$	$P2_12_12_1$	$I4_122$	$I4_122$
Unit cell dimensions	a=60.18Å, b=65.41Å, c=94.82Å; α=β=γ=90°	a=60.56Å, b=65.49Å, c=95.11Å; α=β=γ=90°	a=60.113Å, b=65.612Å, c=94.813Å; α=β=γ=90°	a=b=79.019Å, c=254.467Å; α=β=γ=90°	a=b=80.125Å, c=255.173Å; α=β=γ=90°
X-ray source	DLS[a]-I04	DLS-I02	DLS-I04	DLS-I04	DLS-I03
Wavelength (Å)	0.97250	0.97949	0.97949	0.97949	0.97625
Resolution range (Å)	65.41-1.71 (1.74-1.71)[b]	60.54-1.3 (1.32-1.3)	53.95-1.41 (1.45-1.41)	75.46-2.66 (2.73-2.66)	63.79-2.24 (2.30-2.24)
Multiplicity	5.8 (4.9)	6.0 (5.7)	6.1 (4.7)	12.3 (12.1)	25.4 (26)
I/σ I	21.2 (8.2)	11.4 (5.1)	10.4 (1.3)	7.8 (0.9)	15 (1.3)
Completeness (%)	100 (99.7)	99.2 (98.2)	99.9 (99.3)	100 (100)	100 (100)
R_{merge}	0.050 (0.158)	0.103 (0.367)	0.073 (0.962)	0.218 (4.017)	0.128 (3.419)
R_{pim}	0.023 (0.079)	0.046 (0.166)	0.034 (0.546)	0.067 (1.226)	0.026 (0.694)
$CC_{1/2}$	0.994 (0.975)	0.987 (0.934)	0.999 (0.509)	0.998 (0.631)	0.999 (0.568)
Wilson B factor	12.9	12.5	11.121	46.29	46.87
Total observations	237944 (10418)	560364 (25848)	446531 (24611)	148779 (10500)	523868 (38946)
Total unique observations	41295 (2141)	92810 (4500)	72941 (5257)	12108 (867)	20604 (1497)
**Refinement**					
R-work	0.1389	0.1198	0.1532	0.2339	0.2078
R-free	0.1663	0.1456	0.1831	0.2921	0.2578
RMS (bonds)	0.013	0.011	0.016	0.012	0.012
RMS (angles)	1.24	1.12	1.49	1.47	1.13
Average B-factor	19.4	19.7	18.6	67.6	58
**Ramachandran plot statistics (%)**					
Favored	99.07	99.4	98.39	97.91	98.28
Allowed	0.93	0.6	1.61	2.09	1.72
Outliers	0	0	0	0	0

6. Additional Crystal Structure Discussion

An overlay of the IPR structure with SalR gave an rmsd of 1.04 Å (over 264 residues), indicating high structural similarity. IPR has a core SDR-like structure, with a Rossmann fold domain composed of seven parallel twisted β-strands flanked by four α-helices on the front and three α-helices on the back side (Figure 1a). A SalR-like "flap-like" domain (residues 100-141), mostly predicted in plant reductases, connects α4 with β4 and caps the substrate and cofactor-binding region along with a large loop (residues 265-282) that originates from the core domain. The helix from the flap domain, which caps the active site, has no direct interaction with **3a**, as the side chains are at a distance of >3.8 Å from the substrate.

Compound β-cyclocitral binds to the active site in a different orientation compared to **3a**, tilted outwards with respect to the nicotinamide ring (Figure S6b), with a change in the Glu238 side chain conformation. This positions the C=C bond of β-cyclocitral in an orientation inconsistent with hydride transfer (5.42 Å).

References

[1] A. Ní Cheallaigh, D. Mansell, H.S. Toogood, S. Tait, N.S. Scrutton and J.M. Gardiner, **2016**, *manuscript in preparation*.

[2] A. Fryszkowska, H. Toogood, M. Sakuma, G. M. Stephens, J. M. Gardiner, N. S. Scrutton, *Catalysis Science & Technology* **2011**, *1*, 948-957.

[3] H. S. Toogood, A. N. Cheallaigh, S. Tait, D. J. Mansell, A. Jervis, A. Lygidakis, L. Humphreys, E. Takano, J. M. Gardiner, N. S. Scrutton, *ACS Synthetic Biology* **2015**, *4*, 1112-1123.

[4] a) B. H. Lipshutz, D. M. Nihan, E. Vinogradova, B. R. Taft, Z. V. Boskovic, *Org. Lett.* **2008**, *10*, 4279-4282; b) T. Hirata, S. Izumi, K. Shimoda, M. Haysahi, *J. Chem. Soc. Chem. Commun.* **1993**, *18*, 1426-1427; c) A. F. Regan, *Tetrahedron* **1969**, *25*, 3801-3805.

[5] G. L. Peterson, *Meth. Enzymol.* **1983**, *91*, 95-119.

[6] F. Gorrec, *J Appl Crystallogr* **2009**, *42*, 1035-1042.

[7] G. Winter, C. M. Lobley, S. M. Prince, *Acta Crystallogr D Biol Crystallogr* **2013**, *69*, 1260-1273.

[8] W. Kabsch, *Acta Crystallogr D Biol Crystallogr* **2010**, *66*, 125-132.

[9] P. R. Evans, G. N. Murshudov, *Acta Crystallogr D Biol Crystallogr* **2013**, *69*, 1204-1214.

[10] M. D. Winn, C. C. Ballard, K. D. Cowtan, E. J. Dodson, P. Emsley, P. R. Evans, R. M. Keegan, E. B. Krissinel, A. G. Leslie, A. McCoy, S. J. McNicholas, G. N. Murshudov, N. S. Pannu, E. A. Potterton, H. R. Powell, R. J. Read, A. Vagin, K. S. Wilson, *Acta Crystallogr D Biol Crystallogr* **2011**, *67*, 235-242.

[11] A. J. McCoy, R. W. Grosse-Kunstleve, P. D. Adams, M. D. Winn, L. C. Storoni, R. J. Read, *J Appl Crystallogr* **2007**, *40*, 658-674.

[12] T. C. Terwilliger, R. W. Grosse-Kunstleve, P. V. Afonine, N. W. Moriarty, P. H. Zwart, L. W. Hung, R. J. Read, P. D. Adams, *Acta Crystallogr D Biol Crystallogr* **2008**, *64*, 61-69.

[13] P. D. Adams, P. V. Afonine, G. Bunkoczi, V. B. Chen, I. W. Davis, N. Echols, J. J. Headd, L. W. Hung, G. J. Kapral, R. W. Grosse-Kunstleve, A. J. McCoy, N. W. Moriarty, R. Oeffner, R. J. Read, D. C. Richardson, J. S. Richardson, T. C. Terwilliger, P. H. Zwart, *Acta Crystallogr D Biol Crystallogr* **2010**, *66*, 213-221.

[14] P. Emsley, B. Lohkamp, W. G. Scott, K. Cowtan, *Acta Crystallographica Section D* **2010**, *66*, 486-501.

[15] P. V. Afonine, R. W. Grosse-Kunstleve, N. Echols, J. J. Headd, N. W. Moriarty, M. Mustyakimov, T. C. Terwilliger, A. Urzhumtsev, P. H. Zwart, P. D. Adams, *Acta Crystallogr D Biol Crystallogr* **2012**, *68*, 352-367.

[16] V. B. Chen, W. B. Arendall, 3rd, J. J. Headd, D. A. Keedy, R. M. Immormino, G. J. Kapral, L. W. Murray, J. S. Richardson, D. C. Richardson, *Acta Crystallogr D Biol Crystallogr* **2010**, *66*, 12-21.

[17] R. P. Joosten, F. Long, G. N. Murshudov, A. Perrakis, *IUCrJ* **2014**, *1*, 213-220.

[18] M. Tanaka, R. Bateman, D. Rauh, E. Vaisberg, S. Ramachandani, C. Zhang, K. C. Hansen, A. L. Burlingame, J. K. Trautman, K. M. Shokat, C. L. Adams, *PLoS Biol* **2005**, *3*, e128.

[19] Y. Fukuda, T. Sone, H. Sakuraba, T. Araki, T. Ohshima, T. Shibata, K. Yoneda, *FEBS J* **2015**, *282*, 3918-3928.

[20] H. Ashkenazy, E. Erez, E. Martz, T. Pupko, N. Ben-Tal, *Nucleic Acids Res* **2010**, *38*, W529-533.

[21] Y. Higashi, T. M. Kutchan, T. J. Smith, *The Journal of Biological Chemistry* **2011**, *286*, 6532-6541.

[22] E. M. Davis, K. L. Ringer, M. E. McConkey, R. Croteau, *Plant Physiology* **2005**, *137*, 873-881.

[23] K. L. Ringer, M. E. McConkey, E. M. Davis, G. W. Rushing, R. Croteau, *Archives of Biochemistry and Biophysics* **2003**, *418*, 80-92.
[24] S. McNicholas, E. Potterton, K. S. Wilson, M. E. Noble, *Acta Crystallogr D Biol Crystallogr* **2011**, *67*, 386-394.
[25] E. F. Pettersen, T. D. Goddard, C. C. Huang, G. S. Couch, D. M. Greenblatt, E. C. Meng, T. E. Ferrin, *J Comput Chem* **2004**, *25*, 1605-1612.
